# Editorial: Advancing interventions and therapeutic outcomes for autistic youth: a multidisciplinary perspective

**DOI:** 10.3389/frcha.2025.1697025

**Published:** 2025-09-16

**Authors:** Bethany Oakley, Roberto Canitano, Miguel López-Zamora, Noemi Mazzoni

**Affiliations:** ^1^ Department of Forensic and Neurodevelopmental Sciences, Institute of Psychiatry, Psychology, and Neuroscience, King’s College London, London, United Kingdom; ^2^ Division of Child and Adolescent Neuropsychiatry, University of Siena, Siena, Italy; ^3^ Department of Developmental and Educational Psychology, University of Malaga, Málaga, Spain; ^4^ Department of Theoretical and Applied Sciences, eCampus University, Novedrate, Italy; ^5^ Department of Psychology and Cognitive Science, University of Trento, Rovereto, Italy

**Keywords:** autism, children and young people, intervention, novel therapeutics, outcome

**Editorial on the Research Topic**
Advancing interventions and therapeutic outcomes for autistic youth: a multidisciplinary perspective

## Current status of the field

Autistic people and their families experience barriers in access to health and social care services (1). Despite autistic traits typically becoming recognised within the first two years of life, average age of diagnosis is ∼4–5-years, with many individuals diagnosed even later and into adulthood (1–4). Missed/misdiagnosis is also common, particularly in autistic girls/women (5–7). Accurate and timely diagnosis is pivotal to accessing appropriate interventions, supporting autistic youth to strengthen skills and achieve their full potential (8).

However, the evidence-base for effective, acceptable, and accessible interventions tailored to the specific needs of autistic youth remains limited (9) - both for interventions focused on features “core” to autism that can cause distress (e.g., negative sensory experiences), and those highly co-occurring with autism (e.g., physical/mental health problems). With the aim to advance understanding of factors influencing the effectiveness of different interventions for autistic youth, this Research Topic gathers studies using integrated and multidisciplinary approaches and innovative methodologies in the field.

## Advancing progress

Support delivered in early development may maximise longer-term outcomes (10–12). Currently, the most established early intervention models focus on behavioural change (13), though these are controversial due to their basis in norms for “typical development” [see (14)] and individual outcomes are also highly variable. For instance, Du et al. showed that diverse behavioural interventions may have differential effects on specific developmental outcomes (e.g., social vs. motor) and suggest that clinicians should consider child-specific needs and contextual factors in selecting a therapeutic approach.

Indeed, to progress the field, we need to move towards a more comprehensive and sensitive consideration of individual differences and community needs and priorities in defining what the targets for intervention should be and how their success should be measured (15–17). Integrating perspectives of autistic young people and their parents/caregivers is thus crucial. The work of Carlsson et al. qualitatively investigates the social validity of early intervention. Parents appreciated the naturalistic and local setting of the intervention and their active participation, which provided them with new knowledge and a sense of empowerment. Results further indicated that parents also have diverse needs and value both broader autism education, and the opportunity to focus on more specific understanding of their own child and strategies to support them, as central components of intervention.

These qualitative insights resonate with the increasingly highlighted importance of actively involving parents as a “mediator” in intervention. Consistently, Carta et al. reported that augmenting traditional behavioural interventions with parental support can lead to further gains in parent-rated child outcomes, as well as reducing parental stress. Such findings emphasise the importance of family systems approaches (18), with emerging evidence that child–parent neurodevelopmental similarity can have protective effects on developmental outcomes of autistic children (19), also acknowledging that a high proportion of parents of autistic young people are neurodivergent and/or experience poor mental health themselves (20).

Further extending this holistic view, it is now apparent that autistic traits and commonly co-occurring features interact and evolve dynamically and differently within and between individuals across development (21). Consequently, no single intervention approach will be effective for all individuals, nor at all developmental stages. To address this challenge, “precision healthcare” approaches have become prominent in autism research. Here, Fradkin et al. administered 8 weeks of Transcranial photobiomodulation (tPBM) therapy and assessed its effects using behavioural and EEG measurements. Results showed a reduction of clinical rated autistic traits and changes in brain activity. Although still in its infancy, this work suggests that EEG metrics may serve as a candidate biomarker that could predict who is most likely to benefit from a mechanistically targeted therapy, based on their individual needs and biological profiles, supporting prior findings (22–24).

Alongside biomarker discovery, interest in biopsychosocial models and their clinical implications in the context of autism has recently been reignited (25). In their systematic review, Yang and Li examined the impact of physical activity interventions on restricted and repetitive behaviours, tentatively suggesting that observed effects may act via sensory/regulatory pathways. Interconnection between brain and body has long been established. However, “body” is often neglected in psychological research, particularly limiting progress in the context of neurodevelopmental conditions that are highly co-occurring with physical health problems affecting daily functioning, quality of life, and mortality (26).

## Shaping the future

Taken together, the research themes captured here indicate that next steps in advancing interventions and therapeutic outcomes for autistic youth include identifying and removing barriers to health and social care access to improve earlier provision of support, and reframing intervention targets to align with the needs and priorities of autistic young people and their families. Participatory approaches and involvement of underrepresented voices (e.g., autistic people with co-occurring intellectual disability, those from lower socioeconomic backgrounds) in these efforts is essential (27). Interventions themselves should be adapted to the experiences of autistic people, as differential responsiveness/side effects in autistic vs. non-autistic individuals, and higher effectiveness of autism-adapted vs. standard-of-practice approaches have been highlighted (28–30). Additionally, a holistic approach addressing neurodiversity-affirmative environmental accommodations (31, 32), family support needs, and overall physical and mental health is critical to improving long-term outcomes for autistic youth.
